# Supplementary material for: A qualitative inquiry on drivers of COVID-19 vaccine hesitancy among adults in Kenya
Source: PLOS Glob Public Health. 2024 Mar 28;4(3):e0002986. doi: 10.1371/journal.pgph.0002986 (PMC10977730; doi:10.1371/journal.pgph.0002986)
Supplement: Attachment — Submitted filename: Response to Reviewers_2024.02.13.docx [file pgph.0002986.s001.docx]

## A qualitative inquiry on drivers of COVID-19 vaccine hesitancy among adults in Kenya

Response to reviewer comments

Dear Reviewers

Thank you very much for the helpful comments on our paper. In the report below, we have responded to your input within a table that outlines your comment and summarizes our response.

| **Journal Requirements:** |  |
| --- | --- |
| 1. Please amend your detailed Financial Disclosure statement. This is published with the article. It must therefore be completed in full sentences and contain the exact wording you wish to be published.  a. State the initials, alongside each funding source, of each author to receive each grant. b. State what role the funders took in the study. If the funders had no role in your study, please state: “The funders had no role in study design, data collection and analysis, decision to publish, or preparation of the manuscript.” c. If any authors received a salary from any of your funders, please state which authors and which funders.  If you did not receive any funding for this study, please simply state: “The authors received no specific funding for this work. | The financial disclosure statement has now been amended and reads as follows:  “This work was supported by funding from the International Decision Support Initiative (IDSI) ( EB, SO). Additional funds from a Wellcome Trust core grant awarded to the KEMRI-Wellcome Trust Research Program (#092654) (EB) and the German Academic Exchange Service (DAAD) (SO). The funders had no role in the study design, data collection and analysis, decision to publish, or preparation of the manuscript.” |
| 2. We have noticed that you have uploaded Supporting Information files, but you have not included a list of legends. Please add a full list of legends for your Supporting Information files after the references list. | This has now been included after the references list. |
| 3. . In the online submission form, you indicated that "The data presented in this study are available upon reasonable request from the corresponding author". All PLOS journals now require all data underlying the findings described in their manuscript to be freely available to other researchers, either 1. In a public repository, 2. Within the manuscript itself, or 3. Uploaded as supplementary information. This policy applies to all data except where public deposition would breach compliance with the protocol approved by your research ethics board. If your data cannot be made publicly available for ethical or legal reasons (e.g., public availability would compromise patient privacy), please explain your reasons by return email and your exemption request will be escalated to the editor for approval. Your exemption request will be handled independently and will not hold up the peer review process, but will need to be resolved should your manuscript be accepted for publication. One of the Editorial team will then be in touch if there are any issues. | Public deposition of the transcripts would breach compliance with the approved protocol. During ethical approval, the transcripts were stated would be available publicly upon reasonable request through KEMRI-Wellcome Trust’s data governance committee through the email [dgc@kemri-wellcome.org](mailto:dgc@kemri-wellcome.org). This was because at the time of data collection, it was deemed a sensitive topic, it was paramount not to compromise patient privacy, and allow free participation among the respondents.  We are therefore kindly requesting for this exemption. |
| **Additional Editor Comments:** |  |
| 1. Thank you submitting this important research to this journal. We've had a speedy review process with meaningful comments and suggestions on how to enhance the manuscript. I am recommending major revisions as the collective list of comments is long and cover the whole manuscript. Nevertheless, I believe these points can be addressed well by the author team.  Overall please focus on:  a) the development and use of the conceptual framework- this appears to be an adaptation rather than a new or novel framework. Please also outline why the domains are independent, why these were necessary to distill from existing literature, how they differ in theory from the definitions of each of the 5Cs, and how they link to the 5Cs which are only discussed in the end of the results section and in the discussion. Please also attribute the components that came from prior models, and describe how  b) rationale for purposefully sampling such a low proportion of unvaccinated people- given the nature of the study and the national vaccinations rates presented. Please outline proportions who were partially vaccinated and their decision to not receive any further dose (not including booster shot- i.e 3rd dose of mRNA type of vaccines etc).  c) emergence of new themes or context specific knowledge that can contribute to tailored design and messaging of vaccination programs or any other health service delivery area, and how these findings are relevant especially since COVID-19 is no longer considered a public health emergency. | Thank you for the valuable feedback. The detailed responses are addressed alongside each comment.  Below please find my overall response to your comments:   a) Yes, the conceptual framework is an adaptation from existing literature of both childhood vaccinations and COVID-19 vaccines. This has now been explicitly mentioned. This was deemed appropriate so as to ensure that the conceptual framework was comprehensive. However, during analysis any new emerging determinants outside those of the conceptual framework would be identified.   Given the criticism that vaccine hesitancy has previously neglected psychological states in its definition, the conceptual framework sought to analyse the influence that the determinants of vaccine hesitancy had on one or more of the 5C psychological constructs (page 6, paragraph 3-tracked version). We have now included more details in the data analysis section on how this link was established (page 10-tracked version).  We have also now described the components that came from prior models i.e. the 4 sub-groups of determinants of vaccine hesitancy was largely adapted from WHO SAGE framework, Further, appropriate drivers of vaccine hesitancy identified from other referenced frameworks were also classified into the 4 sub-groups (page 5, paragraph 2-tracked version). We have also revised this to indicate that the four domains of vaccine hesitancy interact. This is now illustrated through arrows in Figure 1 that show the interaction across the four domains.   b) We did not purposefully set out to sample a low proportion of unvaccinated individuals in the study. The low proportion of unvaccinated people is partly due to majority of those unvaccinated not consenting to be part of the study, which is now acknowledged in the limitations (page 29, paragraph 1-tracked version).   However, we included both vaccinated and unvaccinated participants in this study because vaccine hesitant individuals include those who not only outrightly refuse to be vaccinated but also those who were initially uncertain and also delayed in getting vaccinated. During the focus group discussions, it was brought out that even among those vaccinated, some were initially uncertain and changed their minds and for some after receiving the first dose and experiencing side effects they were hesitant to receive any more doses. This time component is brought out in the results and we have now updated the conceptual framework to reflect this (page 7-paragraph 2-tracked version and figure 1).  Participants in the study received multi-type vaccines and most respondents were not knowledgeable on which type of vaccine they had received. Therefore, we did not have sufficient information to ascertain whether they were fully vaccinated (by 2 primary dose vaccine or 1 primary dose vaccine) or partially vaccinated.   c) This study provides context specific knowledge including the need for more gender-responsive immunization programs, the need for targeted messages that are tested in the local context and the need for further-in depth analysis on reasons why awareness campaigns in the local context may have not managed to dispel rumours. Lastly, the need for a multisectoral approach beyond the health sector (including cultural and religious leaders) that leverage on diverse expertise, knowledge, reach and resources towards building trust in vaccines (page 30- last paragraph-tracked version).   While COVID-19 is no longer a public health emergency, this study contributes to the knowledge of drivers of vaccine hesitancy in the local context that can inform preparedness of future pandemics and future routine vaccines rolled out to adults such as the seasonal influenza vaccine (page 29 last paragraph-tracked version). |
| **Introduction**  1. Ln69 &70: “In Kenya, routinely vaccinating adults is a novel area, that has gained much appreciation since the COVID-19 pandemic” What about seasonal Influenza? Perhaps elaborate on trends of access and uptake, including within high-risk populations- and reasons why adult vaccination was considered in the country as ‘novel’. | This has been rephrased for better clarity to read: “Historically in Kenya, routine vaccinations have focused on children and adolescents through the Kenya Expanded Program for Immunization, making adult vaccinations a relatively new initiative, further emphasized by the COVID-19 pandemic” (page 3 last paragraph-tracked version).  The seasonal influenza vaccine was recommended in 2016 for introduction in Kenya among children 6 to 23 months of age. The vaccine was not recommended for any other risk group due to the lack of local burden of disease data (page 3 last paragraph-tracked version). |
| 2. The introduction section will benefit from outlining definitions and literature around adult vaccine hesitancy, contrasting this concept with vaccine acceptance (and discussion around the spectrum). Then discuss specifically for COVID-19 in global and LMIC context, before discussing the Kenyan context. | We have now pointed out what the spectrum of vaccine hesitancy entails. (page 4 paragraph 1-tracked version)  Further, we have discussed COVID-19 vaccine hesitancy in the global, LMIC context before discussing in the Kenyan context (page 5 paragraph 2-tracked version). |
| 3. Which COVID-19 vaccines were available in Kenya during this time? What were specific themes of global hesitancy or concerns on each of these class/types of vaccines? | The available COVID-19 vaccines in Kenya during this time have now been specified and included AstraZeneca, Pfizer, Johnson and Johnsons, Moderna, and Sinopharm vaccines (page 3 paragraph 2-tracked version).  The specific themes of global hesitancy among these vaccines largely focused on safety and efficacy concerns and have been highlighted in the introduction (page 5 paragraph 2 tracked version). |
| 4. As the authors highlight- the COVID-19 is no longer a public health emergency. Please elaborate on why understanding the reasons for COVID-19 vaccine hesitancy is necessary now- perhaps in preparedness for future pandemics? Authors describe its impact on future routine vaccines in lines 85 &6- which adult vaccines? | This has now been rephrased to elaborate that understanding the reasons for COVID-19 vaccine hesitancy is important for preparedness for future pandemics and future routine vaccines rolled out in Kenya to adults such as the seasonal influenza vaccine (page 5 paragraph 2 tracked version). |
| **Methods**   1. Please provide rationale- why was a newly developed (or rather- an adapted version of the 5C psychological constructs) conceptual framework necessary? Is this an analytical framework instead? | The conceptual framework is an adaptation from existing literature of both childhood vaccinations and COVID-19 vaccines. This has now been explicitly mentioned. This was deemed appropriate so as to ensure that the conceptual framework was comprehensive. However, during analysis any new emerging determinants outside those of the conceptual framework would be identified (page 6). |
| 2. Also, why were the four domains of determinants considered independent? | Thank you for pointing this out. We have now revised this to indicate that the four domains of vaccine hesitancy interact (page 7 paragraph 2-tracked version). For example, some individual perceptions may be influenced by the context the individual is in. This is now illustrated through arrows in Figure 1 that show the interaction across the four domains. |
| 3. What about time dimension- did time change people’s perception and decisions? Even when they discussed the pandemic retrospectively? | Yes, individual’s decisions around vaccination may vary across time, as reported in the results across the various determinants of hesitancy. This has now been clearly highlighted in the description of the conceptual framework and in Figure 1. |
| 4. How does the participant demographics in each setting reflect the composition in each of the geographical areas? | In Kilifi, the male and female population proportion is 48% and 52% respectively while in Nairobi it is 50% and 50% respectively. The mean age in Kenya is 19 years. In our study, the participant demographics did not necessarily reflect the composition of the geographical areas as we aimed to purposively sample across the different age groups, gender, socio-economic status, education status and COVID-19 vaccination status. |
| 5. What was the reason for including vaccinated participants in a study about hesitancy- where 80% had at least one dose? How many had 2 doses (fully vaccinated)? Only 16 of all FGD were unvaccinated at time of FGD. Did any of those vaccinated even partially change their minds before first being hesitant, or after receiving the first dose? This speaks about the time component in the conceptual/analytical framework | We included both vaccinated and unvaccinated participants in this study because vaccine hesitant individuals include those who not only outrightly refuse to be vaccinated but also those who were initially uncertain and also delayed in getting vaccinated. During the focus group discussions, it was brought out that even among those vaccinated, some were initially uncertain and changed their minds and for some after receiving the first dose and experiencing side effects they were hesitant to receive any more doses. This time component is brought out in the results and we have now updated the conceptual framework to reflect this.  During data collection of socio-demographic characteristics, we asked the participants 1) Have you received the COVID-19 vaccine? and 2) Have you received the COVID-19 vaccine booster dose?  In the first question, we did not specify whether they had received 2 primary doses or 1 primary dose. This was because participants had received multi-type vaccines and not all participants were knowledgeable on which vaccine type they had received. Therefore, we would not have sufficient information to know how many were fully vaccinated and how many were partially vaccinated, given those who received J&J only needed 1 primary dose. |
| 6. What are characteristics of the 8 healthcare managers and providers? | We have included under the study design and data collection section that they included 4 nurses, 1 community health assistant and 3 county health managers involved in demand generation activities (page 9 paragraph 1-tracked version). |
| 7. In FGDs, what were observations around who spoke, and who were silent? What were group dynamics observed? How did the identity of the data collector likely influence engagement and discussions- especially tying in with social desirability which is discussed in limitations section. | In the FGDs, there were varied observations around who spoke and who tended to be silent, as it was generally a mix of both gender and vaccination status. However, to mitigate this, the data collectors during the sessions would elicit further information from quieter participants and manage dominant participants.  Data collectors were researchers, and this may have influenced the discussions. However, prior to the start of the FGDs, there was an information session where the researchers communicated the aims of the study, that confidentiality of the participants would be maintained and clarified any misunderstandings of the FGDs participants to lessen social desirability among the participants (page 8 last paragraph-tracked version). |
| **Results**   1. Why were results divided based pre-identified determinant from the scoping reviews, and how did the 5C model feature in the coding and analysis? | The results were based on the determinants of vaccine hesitancy primarily. However, given the criticism that vaccine hesitancy has previously neglected psychological states, we sort to also report the influence of these determinants on the 5C psychological constructs.  During analysis we first identified themes in relation to the determinants of hesitancy. Subsequently, we also mapped how these determinants related to any of the 5C psychological constructs, which resulted in the causal loop diagram (described in page 10-data analysis section). |
| 2. What were any new emergent codes and themes that fell outside the pre-established framework, and how do these provide new insights especially in the local context? | The new emergent codes that fell outside the pre-established framework included first, the gender roles in rural settings that influenced vaccine hesitancy. Second, the perceived health status where people with comorbidities expressed more concerns over vaccination. Third, the cultural belief that a disease similar to COVID-19 was in existence previously (“Kivuti”).  These provide new insights in the local context including the need for more gender-responsive immunization programs, the need for targeted messages that are tested in the local context and the need for further-in depth analysis on reasons why awareness campaigns in the local context may have not managed to dispel rumours. Lastly, the need for a multisectoral approach beyond the health sector (including cultural and religious leaders) that leverage on diverse expertise, knowledge, reach and resources towards building trust in vaccines. |
| 3. How did narratives from unvaccinated, partially vaccinated and fully vaccinated participants, including those Booster shots differ? How did the different dosing regimes influence decisions and narratives? Please include this data in the FGD participant table. | Narratives did not differ based on vaccination status. However, the different dosing regimens did influence narratives as highlighted in the results: There was vaccine preferences that influenced vaccine hesitancy among the respondents (page 21 paragraph 1-tracked version). This was largely based on the dosing regimen where most people wanted J&J vaccine as it was only one primary dose. We also report a lack of understanding on the rationale for boosters and the number of booster doses to be given that influenced vaccine hesitancy (page 20 last paragraph-tracked version).  In the FGD participant table, we are not able to report data by unvaccinated, partially vaccinated and fully vaccinated. This is because participants received multi-type vaccines and most of the participants were not knowledgeable on which type of vaccine they had received. Therefore, we did not have sufficient information to ascertain whether they were fully vaccinated (by 2 primary dose vaccine or 1 primary dose vaccine) or partially vaccinated. We therefore resulted to collecting information on whether they were vaccinated (partially or fully) or not. |
| 4. In relation to this- please also provide the proportion of vaccinated, partially vaccinated and fully vaccinated in each FGD group, and how this composition may have influence the discussions by groups. Did unvaccinated people or those chose to be partially vaccinated speak up during group discussion? | As mentioned above, we are not able to report data by unvaccinated, partially vaccinated and fully vaccinated. This is because participants received multi-type vaccines and most of the participants were not knowledgeable on which type of vaccine they had received. Therefore, we did not have sufficient information to ascertain whether they were fully vaccinated (by 2 primary dose vaccine or 1 primary dose vaccine) or partially vaccinated.  Participation in the FGDs was mixed across vaccination status (those vaccinated vs not vaccinated). However, researchers elicited further information from quieter participants and managed dominant participants. |
| 5. I think productivity concerns do not necessarily sit under differences in social demographic characteristics- it can be flipped around to be structural since people who work on daily wage will be missing out if they experience side effects or any longer term consequences in comparison to those with jobs that provide some ‘protections’ i.e- paid sick leave etc. | Productivity concerns have now been rearranged from being reported under socio-demographic characteristics to under structural differences in employment in the contextual influences section (page 14, second last paragraph-tracked version). |
| 6. Gender, - gender roles and gendered patterns of health-seeking even outside vaccinations, is a notable dimension of uptake and hesitancy- not just a sociodemographic factor- but here biological sex plays a role too- in the quotes there is reference to ‘rumours that they would become impotent’ (ln 221)- this is a sex-related concern, where awareness campaigns have not managed to dispel these known fears. These areas require further in-depth analysis… | We have now pointed out in the discussion that there is need for future in-depth analysis on reasons why awareness campaigns may have not managed to dispel some rumours and fears (page 27 paragraph 3-tracked version). We also highlight in the discussion the complexity in tailoring appropriate messaging that also require to be tested in the specific context (page 26 last paragraph-tracked version). |
| 7. Myths, health status- the quote on lines 253-255 is an interesting one- that while this person may be seen as high risk of the negative consequences of infection, they are more fearful of the potential negative consequences of the vaccine. | Yes, this was an interesting finding, and was a new theme in the local finding that could be useful in contributing to tailored messaging for the local context, as highlighted in the discussion. |
| 8. The quote on lines 257 to 262 on use in pregnancy speaks about the time factor- that with time and exposure to new information and seeing other like them experience positively is a driver that can address hesitancy, and it also speaks to the importance of awareness building and right messaging. | Yes, this speaks to the time-dimension that is now highlighted in the conceptual framework (page 7 paragraph 2-tracked version and figure 1). We have also highlighted in the discussion the importance of awareness building and right messaging (page 27 paragraph 1-tracked version). |
| 9. In the introduction, the authors mention adult vaccinations being novel in Kenya, but speak about past experience of other vaccines in the uptake of COVID-19 vaccines, including fear of needles. Please elaborate on the types of adult vaccines that could have led to this, or alternatively with needles- could it also be any medicines administered through syringes? | Here we are referring to the latter, people with fear of needles due to past experiences with other vaccines (childhood vaccines) or other medicines administered through injections. We have now clarified this (page 14 paragraph 2-tracked version). |
| 10. Contextual- discussions in this section relate more to sociocultural influences than context: the role of social media and different sources of information, trusted information source/people- CHV-, religion, faith & traditional knowledge over modern medicines; Here the importance of capacity building and incentivizing CH workers or volunteers is useful to build health systems preparedness and resilience in future crises. | The discussions in this section although may relate to sociocultural influences, are closely linked to the context in which they exist. These factors have also been categorized under contextual influences in line with data from literature reviews.  Yes, we agree that capacity building and incentivizing CHVs is important in building health systems preparedness and resilience in future crises. This has also now been highlighted in the discussion (page 25 paragraph 1-tracked version). |
| 11. The discussion about Kivuti is interesting and is unique to the local context- what is the history of this, and is this discussed in medical anthropology literature? | Yes, this is unique to the local context. However, we have not come across it in medical anthropology literature. There were newspaper articles, during the pandemic that highlighted elders from certain tribes having the cure to COVID-19 as the symptoms are similar to Kivuti. |
| 12. Enforced restrictions as a driver that compelled people to be vaccinated- not necessarily addressed their hesitancy, but they valued their freedom to move, including for work- here another form of productivity concerns is presented. More discussions around policy enforcement as a structural driver is useful but discuss and comment on how it can impact on individual agency a failure to fully address the root of hesitancy. Vaccination is not the same as vaccine acceptance- so it will be important to discuss the influence of mandatory measures here. | Thank you for this. We have included this in the discussion section, in addition to illustrating from literature the impact of mandates in different settings (page 25 last paragraph to page 26-tracked version). |
| 13. Quote on line 365- speaks to vaccination programs and the venues or ease of access being a driver. | Yes, this is correct. We have now explicitly highlighted that ease of access was a driver of vaccine uptake. |
| 14. Myths and rumours- the different narratives around population control, long term negative effects and ‘eradication of elderly’ are interesting to prepare for any future epidemics/pandemics | Thanks for this. We have now included this in the discussion (page 25 paragraph 1-tracked version). |
| 15. The next section is similar- misinformation- how are these different to myths and rumours? | The next section of misinformation is presented differently as it highlights the misinformation/lack of understanding among the respondents on how vaccines work in general (e.g. vaccines are given to prevent diseases in the first place and not to cure, and the rationale for boosters). It was reporting a lack of knowledge on the subject matter (i.e. how vaccines work) and not necessarily linked to myths and rumours. |
| 16. Availability of vaccines types is structural- what influenced this and what was available in these two settings? Any differences? | All the vaccines that were available in the country have been specified in the introduction. However, from time-to-time, there were vaccine stockouts of some of these vaccine types through out the country influenced by supply-side challenges from the different procurement channels. When there were stockouts of a specific vaccine type, this was a country-wide problem and therefore there were no differences between the two settings. |
| 17. In the discussion around vaccine stock or stockouts- please explain why Pfizer was allocated to pregnant & lactating mothers, and why there were stock issues. “Sending them away” speaks to the convenience of access- where lack of stock, and structural factors that influence these barriers prevail. | According to the guidelines from the Ministry of Health in Kenya, pregnant and lactating mothers were only eligible to receive mRNA vaccines. However, as mentioned above, due to supply-side challenges and based on their availability from the different procurement channels, then stockouts of these mRNA vaccines would sometimes happen in the country. |
| 18. Novelty of adult vaccination is context-based theme here: perhaps seasonal influenza shots are not common? Why? There is an interesting reference to polio by FGD participant (ln 466-467). Are there lessons that can be drawn from historical programs? | The seasonal influenza vaccine was recommended in 2016 for introduction in Kenya among children 6 to 23 months of age. The vaccine has not recommended for any other risk group in the country due to the lack of local burden of disease data.  Yes certainly, lessons can be drawn from historical immunization programs including the importance of adequate and consistent sensitization on the importance of the vaccines. This is highlighted in the results. |
| 19. How were confidence, constraints and complacency identified as main antecedents? There is missing description and discussion of this. | We have now included in the conceptual framework and data analysis section that further to coding based on the determinants of vaccine hesitancy, we also mapped how these determinants related/influenced any of the 5C psychological constructs. Based on this, only 3 of the 5 psychological constructs were found to relate to the identified determinants of hesitancy in our context. This influence was illustrated through a causal loop diagram. |
| 20. Feedback loop and framework method- how did the analysis of themes contribute to the creation of the feedback loop? Please provide more details on process. | In the data analysis section (page 10-11 -tracked version), we have provided more information. After coding of the determinants of vaccine hesitancy, we mapped the influence (positive/negative) of the determinants on any of the 5C psychological constructs and their influence on vaccine hesitancy, illustrated through a casual loop diagram. In the casual loop diagram, how the variables influence each other is illustrated using an arrow. A feedback loop is shown when factors interconnect with each other. A balancing feedback loop refers to the counter effect by the interconnected variables while a reinforcing feedback loop refers to more of the effect being generated. |
| 21. In the loop diagram, it appears that you are suggesting theoretical propositions or hypothesis that can be later tested. Is this the case? Please review and discuss how the relationships you propose features in current literature, and how it may be a contribution to research in this area. | In the discussion we highlight that we hypothesis reinforcing feedback loops between the three psychological constructs (confidence, complacency, and constraints) and vaccine hesitancy. Implying that when vaccine hesitancy is high, vaccine confidence is low, individuals are complacent and there are no motivations to address constrains in the system. On the other hand, when individuals have low confidence in the vaccine, are highly complacent and there are many constraints in the system, there is increased vaccine hesitancy. These feedback loops highlight the potential undesirable cycles that can consequently affect coverage level. Similar findings of reinforcing feedback loops between confidence and constraints with vaccine hesitancy are reported in a study from African settings on childhood vaccines. We recommend further work on the relationship between the psychological constructs and determinants of hesitancy, especially for adult vaccines. (page 27 paragraph 2-3 -tracked version). |
| **Discussions:**   1. The results section is divided based on domains from scoping review but proposition, and now discussions is heavily based on 5Cs. | This has now been changed and discussion is now primarily structured based on the determinants of vaccine hesitancy i.e. individual perceptions, contextual influences, and COVID-19 vaccines and related factors highlighting any influences of these determinants on 5c psychological constructs.  This is now in line with how the results section is presented. |
| 2. How are drivers of vaccine hesitancy any more complex in Kenya as opposed to any other country, or globally? (Ln 498) | Drivers of vaccine hesitancy in Kenya are complex, as is the case in other settings. We have now highlighted this and referenced global literature and that from low-and middle-income countries that show this complexity in other settings (page 23 paragraph 1-tracked version). |
| 3. Again, the link to lack of confidence, high complacency and constraints (Ln 499) is unclear. | How the link to the psychological constructs was made has now been expounded on earlier under the data analysis section. |
| 4. The link between vaccine complacency and the lifting of structural restrictions is unclear based on the data and discussions presented in the results section. Again the need to discuss time is important- especially in how it influenced decisions or changing decisions. | In the results, the respondents reported that lifting of restrictions created a perception that COVIID-19 was no longer in existence. This therefore created a perception that vaccination was not necessary (i.e. complacency) among the respondents. Due to this lifting of vaccine restrictions was mapped to influence complacency.  We have now included in the discussion the importance of time coupled with exposure of new information over time and seeing others with positive experiences as a driver than can address hesitancy (page 27 paragraph 1-tracked version). Highlighting the importance of consistent awareness building and right messaging over time. |
| 5. The importance of gender-sensitive programs for vaccination, and those that address work factors i.e. timing, productivity concerns is important- perhaps a national mandate time away from work, or work based vaccination programs? | Thank you for this suggestion. We have include in the discussion the need for gender-responsive immunization programs and considerations for national mandates that allow for time away from work for vaccination during pandemics or work based vaccination programs to address productivity concerns and timings that were drivers of vaccine hesitancy (page 26 paragraph 1-tracked version). |
| 6. 6. Consistency of language is useful- is the focus vaccine hesitancy or vaccine uptake? Ln 561 | This has now been changed to vaccine hesitancy. |
| 7. Limitations- why or what makes the study not generalizable? Indeed vaccine hesitancy varies across time (which further reiterates my point on the need to review the influence of time, or how decisions changed with time- as it is discussed retrospectively at the time of the data collection). How may have social desirability influenced the participants discussions or even the data that they self-report- i.e their vaccination status? | As part of limitations, we state that the study may not be generalizable because 1) of the qualitative nature of the study that provides a contextualized understanding of drivers of vaccine hesitancy 2) vaccine hesitancy is contextual as it varies across time and place.  The results point out the influence of time in vaccination decision making. We have now also included this in the discussion.  Social desirability may have influenced participants to respond in a manner that is viewed favourable by others (researchers or even other participants). As a result, this could have influenced how they self-reported their vaccination status or their narratives on vaccine hesitancy. |
| 8. What specifically does the study contribute to knowledge of vaccine hesitancy in LMICs? | The study contributes to knowledge of vaccine hesitancy by highlighting the drivers of vaccine hesitancy in the local context and pointing out the need for evidence based targeted strategies, gender-responsive immunization programs, appropriate messaging that is locally tested and leverages on community driven concerns, and incorporates a multi-sectoral approach. |
| 9. What are context or regional specific knowledge that requires further research? This could be how traditional remedies are perceived etc. | We have recommended further work to test the relationship between psychological constructs and determinants of hesitancy, especially for adult vaccines. There is also a need for future in-depth analysis on reasons why awareness campaigns in the local context may not have managed to dispel rumours and fears, as well as an in-depth understanding on how traditional remedies are perceived (page 27 paragraph 3-tracked version). |
| 10. Again, while COVID-19 is no longer a public health emergency- what does this knowledge contribute to beyond COVID-18- perhaps in the future or in other health system areas? | While COVID-19 is no longer a public health emergency, this study contributes to the knowledge of drivers of vaccine hesitancy in the local context that can inform preparedness of future pandemics and future routine vaccines rolled out to adults such as the seasonal influenza vaccine (page 29 last paragraph -tracked version). |
| **Reviewer 1** |  |
| Thank you for the opportunity to review this important work. The area of work presented in this paper offers new and valuable insights into global health. Feedback:  1. The conceptual framework referred to in the methods section should be introduced in the Introduction section. This is crucial to establish the main argument at the outset of the paper and to emphasize the primary findings discussed in the results section. This linkage between the introduction and the subsequent sections is currently missing and can be enhanced by conducting a more comprehensive review of the existing literature that forms the basis of the conceptual framework. | Thank you.  We have now included in the introduction section a review of existing literature that exists over the years on different conceptual frameworks that have been proposed to assess vaccine hesitancy. This now forms a linkage to the subsequent section that describes how we adapted the conceptual framework for this study (page 4 last paragraph to page 5-tracked version). |
| 2. The results section is organized around the four determinants of vaccine hesitancy. However, the sub-headings (bolded) and the narrative within the results section sometimes diverge, and it would be beneficial to present the information more concisely and regroup it to clearly highlight the four determinants. | The sub-headings under the four determinants are in-line with what was proposed from other conceptual frameworks. We have re-looked at this to ensure they align well under the four determinants. |
| 3. The discussion section would benefit from providing deeper insights into how this research aligns with global health initiatives promoting vaccine equity (based on the Results section) and discussing strategies for enhancing vaccine campaigns, especially across low- and middle-income countries (LMICs). | We have included in the discussion that this study aligns with initiatives promoting vaccine equity because beyond addressing inequitable vaccine distribution, overcoming vaccine hesitancy is necessary to achieving vaccine equity (page 29 last paragraph-tracked version).  Based on our results, we have recommended further in-depth analysis on reasons why vaccine campaigns in the local context may not have managed to dispel rumours and fears and increase vaccine uptake (page 27 paragraph 3 -tracked version). |
| **Reviewer 2** |  |
| Introduction   1. The introduction successfully sets the global and local context of the COVID-19 pandemic, which provides a backdrop for the study's focus. A research gap in the existing literature is identified, underscoring the need for qualitative studies that offer in-depth insights into vaccination decisions. | Thank you. |
| 2. In line 61, there's a goal mentioned about vaccinating 100% of the adult population by December 2022. Yet, by March 2023, only 36.7% of adults were vaccinated. It might be useful to question more explicitly why, aside from initial supply-side challenges, this ambitious target was missed so significantly. | We have now included that aside from supply side challenges being addressed, low uptake persisted. Implying that demand side factors such as vaccine hesitancy threatens vaccine uptake, with estimates of more than a third of the Kenyan adult population classified as being vaccine hesitant at the onset of vaccine rollout in the country (page 3 paragraph 2-tracked version). |
| 3. The sentence in lines 69-71 regarding the novelty of adult vaccination in Kenya seems a bit indirect. Furthermore, the phrase '...has gained much appreciation…' seems to conflict with the subsequent introduction of vaccine hesitancy. Consider rephrasing for clarity: "Historically in Kenya, routine vaccinations targeted children and adolescents, making adult vaccination a relatively new initiative, further emphasized by the COVID-19 pandemic." | Thank you for the recommendation. This has now been rephrased for clarity (page 3 last paragraph-tracked version). |
| 4. Line 73-75 discusses the definition and complexity of vaccine hesitancy. Consider briefly elaborating on why it was deemed one of the top 10 threats to global health in 2019 to provide readers with context. | We have now included that the prevalence of vaccine hesitancy over several decades and the threat it poses in reversing progress made in addressing vaccine preventable diseases has led to it being listed as one of the top threats to global health in 2019 (page 4 paragraph 2-tracked version). |
| 5. In lines 77-79, Kumar et al.'s societal reactions to vaccinations might benefit from a brief explanation or example so readers can understand what each phase entails without referring to the original source. | We have now included a brief explanation on what each phase entails (page 4 paragraph 2-tracked version). |
| Methods:   1. The section on "Conceptual framework" offers a detailed breakdown of determinants of vaccine hesitancy. The 5C psychological constructs are well-defined. However, elaborating briefly on how these constructs were adapted specifically for this study may add depth. | In the introduction, we have now included a background of proposed conceptual frameworks over the years for assessing vaccine hesitancy.  Later under the section, “conceptual framework”, we have now highlighted that In this study, given the criticism that vaccine hesitancy has previously neglected psychological states and focused on behaviour, we sought to also show the influence of determinants of vaccine hesitancy on the psychological constructs.  Subsequently in the data analysis section we now expound on how the influence of the determinants of vaccine hesitancy on psychological constructs is done. |
| 2. In line 121, ’"they influence on or more of the psychological constructs" seems to have a typographical error. | The typographical error has been corrected |
| 3. While the spectrum from complete refusal to complete acceptance is mentioned, consider detailing more on the intermediate stages. What exactly constitutes "refusal but not certain" or "acceptance but not certain"? Providing examples or scenarios could enhance comprehension. | Refusal but not certain indicate those who initially refuse vaccination but later may change their mind and get vaccinated, as a result there is a delay in getting vaccinated.  Acceptance but not certain indicate those who accept to be vaccinated but remain uncertain on whether a right decision was made to get vaccinated.  This has now been clarified (page 7 paragraph 2-tracked version). |
| 4. Towards the end, it might be useful to add a brief note about the limitations of the conceptual framework or any potential biases that could arise from its use. | We have now included that a limitation of using a conceptual framework in data analysis is that the themes identified may be limited to those in line with the framework. However, to mitigate this, any new emerging themes outside of the conceptual framework would be identified during analysis (page 7 paragraph 3-tracked version). |
| Study settings  1. The section provides a succinct overview of the counties selected for the study. You can offer context as to why it was essential to study urban vs. rural, or high vs. low vaccination coverage. | This has now been elaborated on. This was done to identify any differences that may exist in the different settings (page 7 paragraph 1-tracked version). |
| 2. The term "purposively selected" is used twice. Provide a brief rationale for the purposive selection method used, as readers may not be familiar  with its application in this context. | Purposive selection of counties and sub-counties was done based on pre-specified criterion i.e. urban vs rural and low vs high vaccine coverage. This was in order to identify any differences that may exist in the different settings. We have now specified this. |
| Study design and data collection   1. Ensure consistency in terminology throughout, for example, ‘health care workers’ and ‘health workers’ | We have revised to ensure consistency in terminology. We are now using health care workers throughout the paper. |
| 2. In line 149, the phrase "COVID-19 vaccination status" can be made clearer by specifying whether this refers to individuals who were vaccinated, unvaccinated, or partially vaccinated. | We have now specified that COVID-19 vaccination status refers to a mix of those who were vaccinated, unvaccinated, and partially vaccinated (page 8 paragraph 3-tracked version). |
| 3. The role of community health volunteers (CHVs) is mentioned in lines 145-147. It would be beneficial to briefly explain why CHVs were chosen for this purpose and their credibility in the community. | We have now explained that CHVs were deemed appropriate to contact FGD participants given their ability to easily identify community members based on the pre-specified criteria and their credibility in the community (page 8 paragraph 3-tracked version). |
| 4. The sentence in lines 166-168 repeats the idea of "saturation" twice, which can be redundant. | This has now been revised (page 9 paragraph 2-tracked version). |
| 5. In Table 1, Ensure that the numbers in the "Summary of FGD participants" match the totals from Kilifi County and Nairobi County. | This has been checked and the totals in the summary of FGD participants tally with the totals from Kilifi and Nairobi County |
| Results  1. The organization of results into four determinants of vaccine hesitancy is commendable. This makes it easier to follow and understand the various factors influencing vaccine hesitancy.  Addressing perceptions around comorbidities, pregnancy, and past experiences with vaccinations is relevant in the context of the COVID-19 pandemic. These factors have been topical issues in vaccine uptake discussions globally. | Thank you |
| 2. It might be beneficial to contrast the perceptions from urban vs. rural settings more explicitly. This would highlight the unique challenges and drivers in each setting. | During analysis, the perceptions of drivers to vaccine hesitancy were similar across both urban and rural settings except in two instances that have been explicitly pointed out 1) Gender roles-there was lack of autonomy in vaccination decision making among women in the rural setting. This was not the case in the urban setting 2) In the rural setting, there were cultural beliefs that a similar disease to COVID-19 existed before. i.e. “kivuti”. |
| 3. The information on cultural and religious beliefs influencing vaccine hesitancy is intriguing. Delving deeper into this topic and providing more details would enhance understanding. | The information on cultural beliefs and in particular there being a respiratory disease similar to COVID-19 in the past, that was treated with herbal medication-“Kivuti” is indeed interesting and unique to the local context. However, there is a lack of medical anthropological literature that we can draw from to provide more details on this. |
| 4.There's a bit of repetition in lines 331-332, where "restrictions" is repeated multiple times; it can be phrased concisely. | This has been rephrased more concisely. |
| 5.In line 356, “COVID-19vaccines” is missing a space. | This has been corrected |
| 6. In lines 483/488, the term "casual loop diagram" appears to be a typographical error. | We have corrected the typographical error to read “causal loop diagram” (page 23 paragraph 2-tracked version). |
| 7. In line 490, consider changing "then vaccine hesitancy will be high" to "this leads to increased vaccine hesitancy." | Thank you for the recommendation. We have rephrased accordingly (page 23 paragraph 2-tracked version). |
| 8. While many challenges and concerns are highlighted, there's limited mention of potential solutions or ways the concerns have been addressed, other than CHV efforts. Expanding on measures taken by health officials, NGOs, or local communities would provide a more balanced view. | In addition to the CHV efforts to sensitize community members, we have also highlighted other measures/solutions to address hesitancy that were reported by the study respondents. Including efforts by leaders to recommend vaccination and publicly taking the vaccine as well efforts by health officials and NGOs to reduce geographical and financial barriers. This was through expanding from vaccinating from the health facilities only to mixed approaches that included outreaches and mass campaigns to increase geographic reach, reduce transport costs and reduce waiting times at the facilities. |
| Discussion  The discussion is logically structured with a focus on vaccine hesitancy's key drivers, the role of communication mediums, the impact of mandates, and constraints. The study context and its relevance two years post-vaccine rollout is adequately established.  The parallels drawn with similar studies from other regions strengthen the argument and contribute to the paper's depth. Mentioning social media's role in shaping vaccine perceptions is crucial in today's context, and this section effectively highlights its impact and the challenge of misinformation. | Thank you. |
| There's a typographical error in the beginning: "qualitative fi1ndings" | The typographical error has now been corrected. |
| Consider expanding on the "multisectoral approach." While the inclusion of community health workers and religious leaders is mentioned, further elaboration on how these sectors can collaborate might enhance the recommendations' clarity. | We have now further elaborated that the multisectoral approach should be a deliberate collaboration and engagement of multiple sectors (such as with religious, cultural leaders, civil society) to leverage on diverse expertise, knowledge, reach and resources towards building trust in vaccines (page 25 paragraph 2-tracked version). |
| Given the importance of combating misinformation on social media, consider suggesting specific strategies or collaborations with tech platforms or leveraging community-driven content to spread accurate information. | Thank you for this suggestion. We have now suggested collaborations with tech platforms to spread accurate information that leverages on community-driven concerns as another strategy to address misinformation on social media (page 25 paragraph 1-tracked version). |
| Conclusion  The conclusion is concise and to the point, effectively summarizing the discussion's main points. Consider expanding on the "transparent and consistent communication" recommendation, providing tangible steps or examples. | We have rephrased this to improve on clarity while ensuring conciseness to read as follows: “These strategies could include gender responsive immunization programs, appropriate messaging and consistent communication that target fear, safety concerns, misconceptions and information gaps in line with community concerns” (page 30-tracked version). |
| **Reviewer 3** |  |
| 1. What was the rationale of interviewing healthcare managers and providers, as the objective is to determine "decisions around whether or not to receive COVID-19 vaccine and the reasons behind them", as indicated in the introduction. The data seems hardly used as almost all excerpts were from FGDs. | The rationale of interviewing a few health care managers was to supplement the FGDs by eliciting views from health care workers on community members vaccine hesitancy. This was done in order to ensure no themes were left out.  After analysis, there were no new themes that were brought out by health care workers that were not identified through the FGDs. In reporting of the results, most of the excerpts used were from FGDs because they were more representative quotes from the community members through the FGDs on the themes identified. |
| 2. In the discussion, the author should discuss impact of starting off their qualitative data analysis using a conceptual framework as opposed to letting the data "speak". Was a mixed approach utilized? | We have now acknowledged under the conceptual framework section that the limitation of using a conceptual framework to guide the analysis if that the themes identified may be limited to those in line with the framework. However, to mitigate this, any new emerging themes outside of the conceptual framework would be identified during analysis (page 7 paragraph 3-tracked version). |
| 3. The authors purposively selected Kilifi and Nairobi county, based only on vaccination coverage. Given vaccination is largely contextual, and Kenya has 47 diverse counties, do the views represent vaccine hesitancy in Kenya? | We selected Kilifi and Nairobi counties based on geographical location and vaccination coverage. As pointed out Kenya has 47 diverse counties. Given the nature of vaccine hesitancy, that is contextual, we agree that this study may not be generalizable to the entire Kenya. This has now been pointed out explicitly in the discussion (page 29 paragraph 1-tracked version). |
| 4. The distribution of the 8 IDIS is broadly defined as health workers and county managers. Would good to specify which cadres and in what quantity of the 8 respondents given the demographics table is not included. Line 156/7. | This has now been specified. IDIs participants included 4 nurses, 1 community health assistant, and 3 county health managers involved in immunization activities (page 9 paragraph 1-tracked version). |
| 5. In the introduction the authors reported that Kenya had a vaccination rate of 36.7%, and specifically indicate that selected counties had vaccination rates of 57.1% and 21.3%. However, the results indicate that 80% of the respondents were vaccinated, with a good majority getting the booster dose. Despite this being a qualitative study, collecting views on vaccination hesitancy should aim to balance those with the outcome, and at the least should have purposively selected based on vaccination status to at least a 50:50 ration to get feedback from majority who did not get vaccinated. This sample is the voice of largely vaccinated people. What dot he unvaccinated (majority in the   sing HCW to recruit study participants? | The sample is largely vaccinated people. However, vaccine hesitant individuals do not only include those who outrightly refuse to be vaccinated but also those who were initially uncertain and delayed in getting vaccinated. Vaccine hesitancy varies across time and some of those vaccinated were initially hesitant at some point.  During recruitment, we set out to get 50:50 ratio of those vaccinated and those not vaccinated. However, participant consent to the study among those unvaccinated was lower. This has now been included in the limitations of the study (page 29 paragraph 1-tracked version). |
| 6. The findings indicate that there was a gender influence on vaccine uptake, especially in the rural area selected, driven by socio-cultural factors. However, it is noted that all FGDs were mixed gender as opposed to gender specific. Do the authors feel that they have accurately captured the weaker gender's perception and drivers to vaccination? What safeguards were in place to ensure free and open expression? | Prior to data collection, this was a concern that the authors had. It was however, established through informal communications with community members and CHVs that for the topic of interest, gender differences would not inhibit freedom of expression but rather age differences was more of a concern.  During data collection, we had safeguard measures to ensure free and open expression including 1) using mixed gender moderators 2) eliciting further information from quieter/shy participants 3) managing dominant participants.  Given the above measures, we feel that we have accurately captured the weaker gender’s perception of drivers to COVID-19 vaccination. |
| 7. In the discussion, kindly be sure to tie it to your findings which you seem to have used various lenses. Started off with the conceptual framework with 4 key constructs, once the themes were developed they were further classified based on the 5C model. Hence the discussion should align with whichever framework or model the author would prefer to present, or clearly present both, so that the content is easier to digest. | This has now been changed and the discussion is now primarily structured based on the determinants of vaccine hesitancy i.e. individual perceptions, contextual influences, and COVID-19 vaccines and related factors highlighting any influences of these determinants on 5c psychological constructs.  This is now in line with how the results section is presented. |
